# Artificial light at night may decrease predation risk for terrestrial insects

**DOI:** 10.1098/rsbl.2022.0281

**Published:** 2022-11-09

**Authors:** Gregory M. Eckhartt, Graeme D. Ruxton

**Affiliations:** School of Biology, University of St Andrews, St Andrews KY16 9TH, UK

**Keywords:** artificial light at night, light pollution, insect predation, insect decline, terrestrial insects

## Abstract

Artificial light at night (ALAN) is thought to be detrimental for terrestrial insect populations. While there exists evidence for lower abundance under ALAN, underlying mechanisms remain unclear. One mechanism by which ALAN may contribute to insect declines may be through facilitating increased predation. We investigated this by experimentally manipulating insect-substitute abundance under differential levels of light. We used insect-containing birdfeed placed at varying distances from streetlights as a proxy for terrestrial insects, inspecting the rate of predation before and after dusk (when streetlights are, respectively, off and on). We found that there was a significantly greater effect of increasing distance on predation after dusk, suggesting that predation was actually reduced by greater levels of artificial light. This may occur because ALAN also increases the vulnerability of insectivores to their own predators. Implications for foraging behaviour and alternative explanations are discussed.

## 1. Background

Insect populations are declining rapidly globally [1]; one meta-analysis estimated a 45% average decline across two-thirds of a global range of evaluated taxa [2]. Artificial light at night (ALAN) is widely regarded as a key stressor contributing to this decline, particularly with regard to the disturbance of trophic processes [1,3–5]. In otherwise dark night-time environments, ALAN attracts both insects and insectivores [6–9] and disrupts insects' defences against predation [10,11].

While global insect defaunation is thought to be partly a result of such mechanisms, the relationship between invertebrate abundance and ALAN is complex; evident in the mixed results of studies to date. While a recent study of terrestrial insects in hedgerows and grass margins found significantly lower abundance in habitats illuminated by streetlights than those not [12], further studies show correlations which are positive [13,14], species dependent [15–17] or non-significant [18]. Possible explanations include varying predation under ALAN.

Two studies found greater predation of terrestrial insects in rural environments after ALAN was experimentally introduced [14,19], however, whether these effects would remain long-term are unknown. One study used established urban streetlights as the source of ALAN, but despite high statistical power found no effect of proximity to streetlights on predation on pinned insect larvae. It may be, however, that effects were masked by a protocol that replaced predated larvae whenever discovered, potentially leading to systematic variation in the freshness of baits available at any given time.

Here, we close knowledge gaps by assessing the effects of long-established sources of ALAN in an urban setting on the predation of terrestrial insects. We propose that, while some evidence points to an increase in terrestrial insect predation under ALAN, it could be that illumination makes insectivores in a terrestrial environment vulnerable to predation themselves, and so ALAN may create a form of ‘foraging exclusion zone’ that protects insects. Thus, predation may actually be lower under higher levels of ALAN, or alternatively, predation may be unaffected by ALAN.

## 2. Methods

### (a) Artificial lighting

To investigate the effects of varying levels of ALAN on the predation of terrestrial insects, we used existing LED streetlight lampposts situated around St Andrews (UK). We determined using a luxmeter during winter at 01.00 that for such lampposts, the brightest point at ground level was directly below the filament, at an average 14.3 lux (*σ* = 0.6), while ambient light levels of 0 lux were reached approximately 6 m from the lamppost. As such, it was determined that a linear vector of nine paces from a lamppost, approximately 7.4 m, was sufficient for light levels to vary greatly at night. With this in mind, we selected lampposts for testing where a consistent grass substrate could be found behind the lamppost for a vector of that length, unimpeded by obstacles and significant change in height, and unintruded by other light sources.

### (b) Artificial prey

In order to simulate terrestrial insects, pre-made insect suet pellets (of the type commonly offered by householders as food for wild birds) were used, which contain a mixture of suet, sugar and insects. Similar artificial prey have been employed in experiments to estimate terrestrial insect predation (e.g. [20–22]).

### (c) Experimental protocol

**Figure 1.**
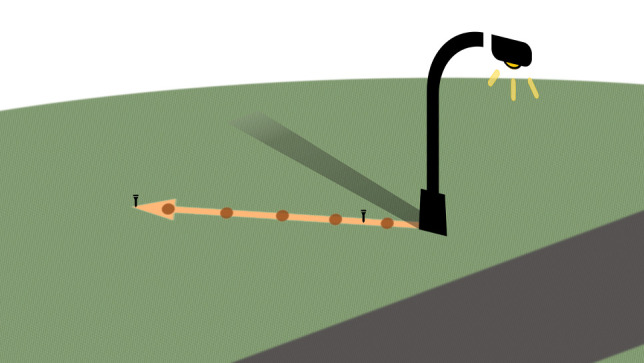
Experimental procedure. An example lamppost with a bait vector is depicted along the orange line, with red circles depicting bait placement, golf tees placed at the end and between the first and second baits, and a dark bar depicting the shadow created directly behind the lamppost.

Data were collected by one experimenter (G.M.E.) over 13 days during the winter and early spring months, January to March 2022, on days where little to no rain and minimal wind was anticipated. To estimate predation levels at varying levels of ALAN, prey were introduced at varying distances across a linear vector behind seven focal lampposts. At each lamppost, five sets of 3–4 insect suet pellets (approximately 0.5 g in total) were dropped from a consistent height directly onto the grass along a straight vector behind the lamppost, first at one pace from the lamppost (each pace of the experimenter was approximately 9/11 of a metre), and then at two paces from one another, avoiding the shadow created directly behind the lamppost (figure 1). Each series of five sets of pellets at each lamppost constituted one ‘batch’, within which, differences in predation could be measured at each distance. Inherent in this design, is the fact that in each after-dusk batch, there were baits set at a distance where illumination was sufficiently low at ground level to mimic ambient light levels, thus effectively forming our dark control. Groups of pellets accounted for minor inconsistencies in the size of individual pellets and aided identification for recovery.

Baits were left out for a 6 h period before dusk (when streetlights turn on), and for a 6 h period after dusk. To allow one experimenter to visit all sites, some were processed a little (no more than 15 min) after dusk (defined by local sunset—closely correlating with onset of streetlight illumination) and some a little before—but care was taken to balance these imprecisions such that no lamppost was visited systematically early or late. We replaced both present and absent baits between time periods. Consistent with previous experiments (e.g. [14,22,23]), a predation event was scored when two or fewer pellets remained. We assigned a binary value (1 or 0) to predation or none. To aid the recovery of baits, two golf tees were placed along the vector, one at the end and one between the first and second bait, indicating the direction of the vector from the lamppost (figure 1).

### (d) Statistical analysis

Data were analysed in R v.4.0.4 [24]. Simple predation rates were initially calculated for the observed levels of predation and tested in pairs of conditions using proportion *χ*^2^-tests [25]. These provide a descriptive outline of the results, but are unable to account for the influence of simultaneous fixed and random effects. As such, the main effects were assessed using a binomial (logit) generalized linear mixed-effects model (GLMM) with the package lme4 [26] and via post hoc pairwise Tukey's tests [27] of the marginal mean responses at each of the five distances at each time period, estimated using the emmeans package [28]. In the final model, we included distance from lamppost, time of day and the interaction there-between as fixed effects. Lamp ID was included as a random factor to account for anticipated differences between lampposts, as we are interested in the effect of distance and time of day within lampposts. The inclusion of batch, nested within lamp ID, as a random factor, accounts for the potential non-independence of predation events within batches. Day was dropped from the final model due to correlating highly with batch. Akaike information criterion and area under the corresponding receiver operating curve (AUC) were used to compare the goodness-of-fit of models employing alternative link functions, confirming that the logit link best fit the data [29–31]. The model was adjusted to investigate the interaction effects, incorporating distance as a discrete factor (as opposed to continuous) to facilitate marginal mean estimation between distances.

## 3. Results

Of 830 prey, 516 were predated across the pre- and post-dusk conditions. Of these, 345 were predated pre-dusk at a predation rate of 83%. The remaining 171 were predated post-dusk at a rate of 41% $(\chi_{1}^{2}=153\;,p<0.001$; further pairwise proportion *χ*^2^-tests can be found in electronic supplementary material S1).

**Figure 2.**
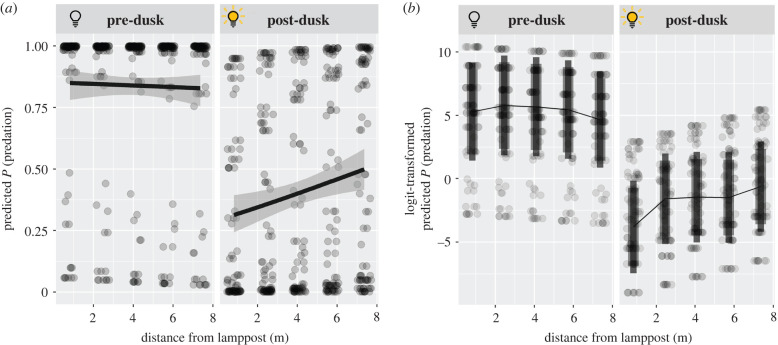
Jitter plots of predicted probability of predation by distance from lamppost before and after dusk. (*a*) Response scale with a glm fit overlayed and 95% confidence ribbons. (*b*) Logit scale with lines displaying estimated marginal means at each distance. Larger vertical bars represent 95% confidence intervals; smaller bars represent 95% prediction intervals. Predation increases significantly after dusk, but not before.

Overall, when accounting for random effects in the full model, predation was significantly greater before dusk than after (GLMM, effect of time category in full model: *z*_824_ = 6.492, *p* < 0.001, table 1). Furthermore, predation rate was significantly higher at greater distances from the lamppost (GLMM, effect of distance: *z*_824_ = 4.290, *p* < 0.001). Importantly though, there was a significant interaction between dusk condition and distance (GLMM, interaction effect of time and distance: *z*_824_ = −3.323, *p* < 0.001, figure 2).

**Table 1.** Results of the main logistic GLMM with rate of predation as the response variable, left out after dusk as opposed to before.

fixed effects	estimate	s.e.	d.f.	*z*	Pr(>|z|)
intercept	−3.320	1.711	824	−1.941	0.052
dusk (pre-)	9.263	1.427	824	6.492	<0.001
distance	0.384	0.089	824	4.290	<0.001
distance * dusk (pre-)	−0.491	0.148	824	−3.323	<0.001
random effects	variance	s.d.			
lamp ID	17.02	4.126			
batch	12.92	3.594			

Marginal means were estimated for predation at each distance in each condition, which revealed that, while predation did not differ with distance to the lamppost significantly before dusk (comparison of predation at each distance before dusk using pairwise Tukey's tests; *p* > 0.95, table 2, figure 2) predation increased significantly between the closest and each further distance after dusk (after dusk between positions 1 and 2, 3, 4 and 5, Tukey's tests; *p* < 0.05). Beyond distances of approximately 2.5 m, predation rates did not differ significantly between distances (Tukey's tests, *p* > 0.75). This test also reaffirmed the finding that predation was significantly greater before than after dusk, with estimated marginal mean predation being greater before dusk at every distance (Tukey's tests, *p* < 0.01). The main model provided a strong level of discrimination, displaying an AUC of 99%.

**Table 2.** Summary of pairwise Tukey's tests comparing estimated marginal means of predation rate at each of the five distance conditions within and between dusk conditions. Only *p*-values are displayed; the full matrix and test statistics can be found in electronic supplementary material S1. Asterisks denote significance at *α* = 0.05*, 0.01** and less than 0.01***.

dusk condition	comparison	distance from lamppost (m)		Tukey's test *p*-value
post	within dusk condition	0.82	2.46	0.023*
post	within dusk condition	0.82	4.09	0.008***
post	within dusk condition	0.82	5.73	0.012**
post	within dusk condition	0.82	7.36	<0.001***
post	within dusk condition	2.46	4.09	1.000
post	within dusk condition	2.46	5.73	1.000
post	within dusk condition	2.46	7.36	0.791
post	within dusk condition	4.09	5.73	1.000
post	within dusk condition	4.09	7.36	0.940
post	within dusk condition	5.73	7.36	0.897
pre	within dusk condition	all combinations		>0.950
both	between dusk condition	all combinations		<0.010***

## 4. Discussion

Light pollution may be a key contributor to global insect declines and consequently the breakdown of vital ecosystem processes. It is thus of paramount importance to understand the nature of this interaction. We found that, in contrast with prevailing thought, predation of surrogates of terrestrial insects was significantly higher at greater distances from artificial light sources at night, and thus at lower light levels. We did not find this distance effect before dusk, when streetlights are not illuminated and thus luminance is spread approximately evenly across the same space. This strongly suggests that the effects we found after dusk are a direct cause of ALAN.

The effects of ALAN varied greatly by lamppost and location, with some experiencing high levels of predation across time and others receiving almost no predation after dusk. This may be indicative of preferential foraging sites or differential predator abundance or activity, although no obvious reason for this difference could be discerned by visual inspection of the sites. Generally, each site contained bushes and grass margins where birds had been seen foraging (G.M.E., pers. obs.). Birds are likely to have been the most active predators at these sites and likely to have been most active during the day, when we indeed found far greater predation rates overall in the 6 h before dusk. Nevertheless, the design of the present experiment did not allow for identification of the predators at each predation event. It should be noted that bats and small ground-dwelling mammals might also have constituted predators of our terrestrial insect substitutes [32,33]. While we are able to show a high-level trend, we are unable to disentangle differences in predation rate across various predators of terrestrial insects. This is a question that future studies may seek to answer by means of cameras or by isolating specific prey and predators; for example, see insectivory by Gastropoda and Coleoptera in [19].

In the present experiment, we isolated changes in the foraging behaviours of predators by controlling for insect behaviour. In this way, we were able to lend power to our investigation of a single causative factor in insect declines under ALAN. In the absence of such controls, insect abundance and defensive behaviour is likely altered by positive phototaxis [10,11,14,34–36]. Future studies might seek to examine the interaction between insect behaviour and predation on insects.

Although anthropogenic disturbance might also vary with distance from streetlighting [37–39], any effect on predation would likely be greater before dusk, when we found no effect of distance. We believe that our results reflect a disturbance effect of ALAN on foraging. Insectivores foraging in a terrestrial environment tend not to be apex predators, instead sitting at an intermediate trophic level, such that top-down predation pressures are likely to significantly influence their foraging behaviour [40]. Crypsis is a key means of defence in the minimization of such predation pressures [41]. The effectiveness of many forms of crypsis are influenced by the nature of illumination [41,42]. It follows then, that for predators of terrestrial insects, in the trade-off between foraging and predation risk, the avoidance of light at night may outweigh the benefits of using light to forage. Thus, ALAN may create a ‘foraging exclusion zone’ for these animals. Indeed, there exists some experimental evidence in support of this notion [43].

In conclusion, despite suggestions that ALAN contributes to lower terrestrial insect abundance [12], we have shown that this may not be due to enhanced predation. It is likely that other mechanisms are playing a role in this case. Our results also suggest potential disturbance effects of ALAN on the foraging behaviour of insectivores. This disturbance may have consequences for trophic ecosystem processes on a global scale, inviting questions for further study. Not least of these questions is which animals are most affected, and whether ALAN is contributing directly to the decline of endangered species. Ultimately, it is hoped that the present study contributes to our understanding and implementation of ALAN in the face of ongoing urbanization.

## Data Availability

Data and data description are available via Dryad Digital Repository: https://doi.org/10.5061/dryad.7m0cfxpxv [45]. Electronic supplementary material S1 contains tables detailing descriptive proportion chi-square tests and the full matrix of Tukey's tests of the estimated marginal means of predation according to a model with distance as a discrete variable [46].
